# Eye tracker calibration: How well can humans refixate a target?

**DOI:** 10.3758/s13428-024-02564-4

**Published:** 2024-12-19

**Authors:** Ignace T. C. Hooge, Roy S. Hessels, Diederick C. Niehorster, Richard Andersson, Marta K. Skrok, Robert Konklewski, Patrycjusz Stremplewski, Maciej Nowakowski, Szymon Tamborski, Anna Szkulmowska, Maciej Szkulmowski, Marcus Nyström

**Affiliations:** 1 Experimental Psychology, Helmholtz Institute, Utrecht University, Utrecht, The Netherlands; 2 Lund University Humanities Lab and Department of Psychology, Lund University, Lund, Sweden; 3 Tobii AB, Danderyd, Sweden; 4 Institute of Physics, Faculty of Physics, Astronomy and Informatics, Nicolaus Copernicus University in Toruń, ul. Grudziadzka 5, 87-100 Toruń, Poland; 5 Inoko Vision, ul. Mickiewicza 7/17, 87-100 Toruń, Poland; 6 Lund University Humanities Lab, Lund University, Lund, Sweden

**Keywords:** Pupil-CR eye tracker, Calibration, Accuracy, Pupil size artefact, Retinal eye tracker

## Abstract

Irrespective of the precision, the inaccuracy of a pupil-based eye tracker is about 0.5$^\circ$. This paper delves into two factors that potentially increase the inaccuracy of the gaze signal, namely, 1) Pupil-size changes and the pupil-size artefact (PSA) and 2) the putative inability of experienced individuals to precisely refixate a visual target. Experiment 1 utilizes a traditional pupil-CR eye tracker, while Experiment 2 employs a retinal eye tracker, the FreezeEye tracker, eliminating the pupil-based estimation. Results reveal that the PSA significantly affects gaze accuracy, introducing up to 0.5$^\circ$ inaccuracies during calibration and validation. Corrections based on the relation between pupil size and apparent gaze shift substantially reduce inaccuracies, underscoring the PSA’s influence on eye-tracking quality. Conversely, Experiment 2 demonstrates humans’ precise refixation abilities, suggesting that the accuracy of the gaze signal is not limited by human refixation inconsistencies.

## Introduction

The vast majority of modern eye-tracking studies are performed with a video-based eye tracker. Video-based eye trackers contain one or more cameras that film the eye. The recorded eye images play a crucial role in gaze estimation. In a first step, objects in the eye image (e.g., pupil, corneal reflection, fourth Purkinje reflection, or outer iris border segments) are extracted from the eye image by means of image processing techniques. These features are the basis for gaze estimation. In the case of a simple pupil minus corneal reflection eye tracker (e.g., SMI Hi-Speed 240, SR Research EyeLink 1000), the features used are the pupil center and corneal-reflection center in image coordinates. From here on, we refer to a pupil minus corneal reflection eye tracker as a pupil-CR eye tracker. In order to deliver meaningful gaze data, in a second step, a transformation from eye image coordinates to screen or world coordinates is required. The transformation itself may consist of a set of mathematical equations (e.g., a second-order approximation) or a biophysically plausible model with several free parameters (e.g., the curvature of the cornea, Barsingerhorn et al., 2018). The parameters for the transformation can be delivered by a calibration procedure. There are various procedures for the calibration, the most common being that the participant fixates a number of calibration markers with known positions. The number of calibration markers may vary per system (one, Niehorster et al., 2020b; five, Hessels et al., 2016, nine, Burggraaf et al., 2018, thirteen, Hooge et al., 2015).

After calibration, a procedure known as a validation could be conducted to evaluate whether the calibration was successful (e.g., SR Research, 2009; Niehorster et al., 2020a, p.30). The participant is again asked to fixate a few validation markers. Ideally, the estimated fixation locations (of the participant’s calibrated eye-tracking data) should coincide with the validation markers. This is usually not the case. The locations of the fixations may be shifted in any direction with respect to the validation markers. To quantify the error, one can calculate the inaccuracy. The inaccuracy can be operationalized as the mean distance between the validation marker(s) and the fixations position(s) (Holmqvist et al., 2012).

There are various ways in which a validation can be conducted. The number of validation targets, the location of validation targets (a large or a small grid), the method for selection of fixation samples (Holmqvist et al., 2015) and the light conditions in the experimental room (Drewes et al., 2014; Holmqvist et al., 2015) may vary. All of these factors may influence accuracy. Manufacturers usually determine eye tracker accuracy under optimal conditions for their product. The optimal conditions may be different for each eye tracker. Some eye trackers perform optimally when using a chinrest while other eye trackers perform optimally under specific lighting conditions of the room (e.g., ideal illuminance is around 300 lux for modern Tobii eye trackers). What the manufacturers report may be treated as the upper limit of accuracy. The manufacturer-reported accuracies make direct comparison between eye trackers difficult because the conditions of the validation may be different. Holmqvist et al. (2015) tried to solve the comparison problem by estimating accuracy of twelve eye trackers under more standardized conditions in 167 participants. By using similar conditions for each eye tracker (e.g., always a 7 by 7 validation grid but on different screen sizes, same room illumination and use of a chin rest), a new problem was introduced, namely that in this test eye trackers may be tested under suboptimal conditions and may deliver worse values than the manufacturer-reported accuracies. Another complicating factor for comparison is that eye trackers are designed to operate under different regimes. Some eye trackers require a fixed head (SMI Hi-Speed 1250, EyeLink 1000 not in remote mode), others are specialized in moderately free heads (remotes such as the Tobii TX300 and LC technologies EyeFollower) and some allow free moving observers (wearables, not in the test of Holmqvist et al. (2015)).

**Fig. 1:**
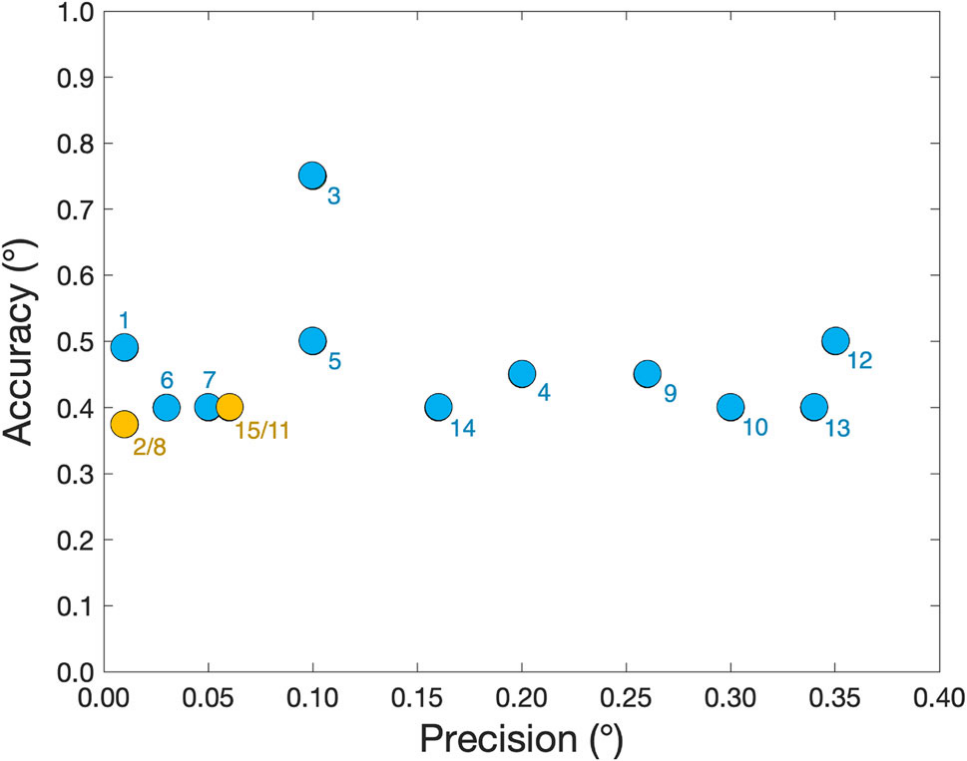
**Data quality for 15 eye trackers**. Accuracy versus precision for 15 pupil-CR eye trackers. The specifications are retrieved from the eye-tracking manufacturers’ product descriptions. SR Research EyeLink 2 [1], SR Research EyeLink 1000 Plus [2], The EyeTribe [3], Eyegaze Edge [4], SMI REDm 120 [5], SMI RED 250 [6], SMI REDn [7], SMI Hi-Speed 1250 [8], Tobii Pro Spark [9], Tobii Pro Fusion [10], Tobii Pro Spectrum *unfiltered* [11], Tobii T60XL [12], Tobii X2 60 [13], Tobii T120 [14], Tobii Pro TX300 *unfiltered* [15]. The *orange dots* represent two eye trackers with similar data quality (2 and 8; 11 and 15). The accuracy of these eye trackers is about 0.45$^\circ$ and from this figure it is clear that the accuracy and precision of the signal of an eye tracker are not related

We are interested in the relation between the precision and accuracy of pupil-CR eye trackers. Precision is the variability in eye-tracking data during fixations and it is usually operationalized as the RMS sample-to-sample deviation (Holmqvist et al., 2012; Niehorster et al., 2020c). A naïve expectation of the data quality of an eye tracker could be that more expensive eye trackers produce data with better precision and better accuracy. Figure 1 depicts accuracy versus precision for 15 different pupil-CR eye trackers. We used the data quality metrics reported by the manufacturers. However, we are aware that the manufacturers’ values can be better than what can be obtained in a lab, however, this is not a problem for the comparison of the relation between accuracy and precision between eye trackers. The precision ranges between 0.01$^\circ$ and 0.35$^\circ$. The accuracy values range between 0.3$^\circ$ and 0.75$^\circ$. Crane and Steele (1985) already wrote about the limit of accuracy in eye trackers using the pupil for gaze estimation:

“A different method for distinguishing between the translational and rotational components of eye movement is based on measuring the position of the corneal reflection with respect to the eye pupil. The advantage of this approach is that the instrument can be located relatively far from the subject. However, the pupil is not a stable reference, and accuracy is limited to 30 min of arc."

Thirty minutes of arc equals 0.5$^\circ$ and is close to the accuracies reported in Fig. 1. That figure also clearly shows that accuracy and precision are not related. Eye trackers with good precision (the left side of Fig. 1) do not have better accuracy values than eye trackers with poor precision. Why is that? Precision may be more related to the eye tracker hardware components (e.g., lens, sensor, and electronic components). Accuracy may depend more on human behavior during calibration and validation. There are at least two potential ways human behavior may affect accuracy.

1. *The pupil-size artefact (PSA)*. When the pupil constricts or dilates, the pupil center may move with respect to the eyeball (Wyatt, 1995). Most pupil-CR eye trackers use the pupil center in their gaze estimation method. Pupil constriction or dilation may thus cause apparent gaze shifts in the absence of eyeball rotation (Wyatt, 2010; Wildenmann & Schaeffel, 2013; Drewes et al., 2014; Choe et al., 2016; Jaschinski, 2016; Hooge et al., 2019, 2021). Consequently, if pupil-size changes occur between and during calibration and validation, accuracy may become worse. We refer to this as the *pupil-size artefact* hypothesis.
2. *Inconsistent fixation during calibration and validation*. Hooge et al. (2021) hypothesized that inaccuracy is mostly due to the inability of the participants to exactly repeat fixation of the calibration and validation targets. They concluded that from the inaccuracies obtained in one of their experiments. In one condition, they calibrated the eye tracker ten times (before each of ten trials). In another condition, they calibrated the eye tracker only once and then recorded with ten trials. The range of inaccuracies obtained in condition one (ten calibrations, see their figure 1) was much larger than the range of inaccuracies obtained in condition two (only one calibration, see their figure 2). A way to interpret this, is that each calibration can be seen as a drawing from the distribution of possible inaccuracies. According to this view one should not recalibrate without a good reason (e.g., some form of drift in the eye tracker signal or slippage of the eye tracker). We refer to this as the *Inconsistent fixation* hypothesis.

**Fig. 2:**
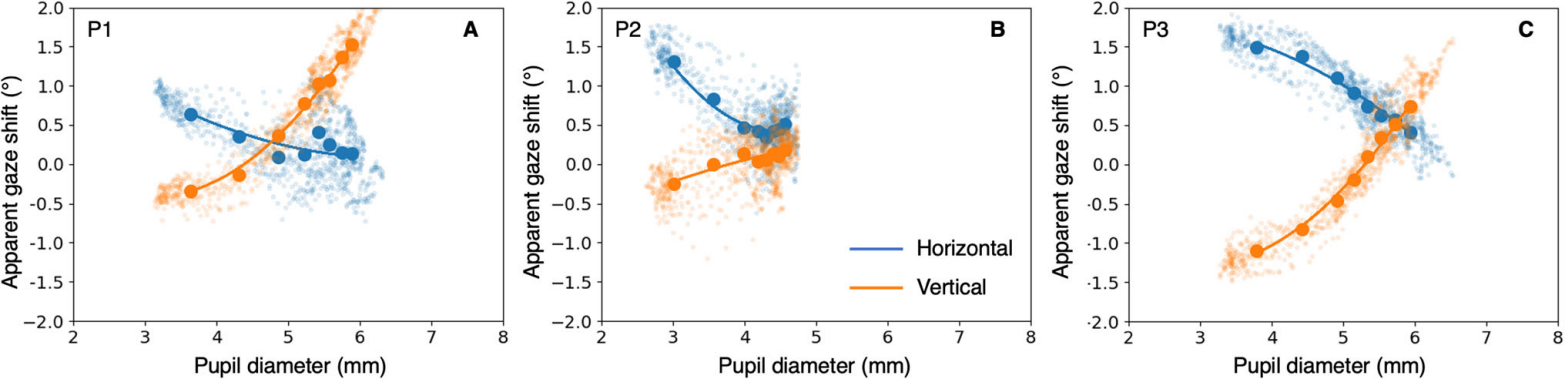
**The pupil size artefact**. This figure illustrates the horizontal (*blue*) and vertical component (*orange*) of the pupil size artefact (PSA) for three participants. We depict the *apparent gaze shift–pupil diameter* relation for each sample (*small dots*). The *larger dots* represent the second until the ninth decile of pupil diameter values. The *lines* represent a second-order polynomial fit to the large dots. On top of that we depict averaged data. Each line consists of eight larger dots, *x*-values representing the second until the ninth decile of pupil size with steps of one-tenth, *y*-values represent the mean of the corresponding apparent gaze-shift values. The *line through the points* is a second-order polynomial fit and is later used for correction of the refixation positions. P1 and P3 exhibit significantly greater variability in their pupil diameter compared to P2, who has smaller pupils. The PSA strengths of the vertical signals from Participants 1 and 3 are the strongest (steepest slope). Participant 2 scarcely exhibits vertical PSA (the slope is close to zero)

We conducted two experiments with two different eye-tracking setups to investigate the putative roles of the pupil-size artefact and inconsistent fixation in causing inaccuracies during calibration and validation. The first experiment uses a regular high-quality pupil-CR eye tracker (SR Research EyeLink 1000 Plus). This is an eye tracker that may be inaccurate due to the PSA (Drewes et al., 2014; Choe et al., 2016; Hooge et al., 2019, 2021). The second is the FreezeEye tracker, a very precise and accurate retinal eye tracker (Bartuzel et al., 2020; Ziv et al., 2022). The FreezeEye tracker is calibration free and uses retinal scans instead of the pupil to estimate gaze.

## Experiment 1: Calibration, validation and the PSA

The goal of the first experiment was to identify the roles of the pupil-size artefact on the time scale of a calibration or a validation (5–20 s). If the pupil changes size for each new fixation, an inaccuracy (due to the PSA) is added to each fixation position. This may affect the quality of both the calibration and validation, and therefore increase the inaccuracy of the eye-tracking data. This was investigated in two experimental conditions. In the *refixation condition*, we calculated the dispersion of refixation positions associated with a specific target. In the *PSA-estimation condition* we estimated the relation between pupil diameter and apparent gaze shift. The apparent gaze shift is the shift in the gaze position that is caused by the pupil size change of a static eye (Wyatt, 2010). Pupil size was manipulated by varying the illumination. Changing the screen from black to white is a simple manipulation to elicit a large pupil size range. According to Drewes et al. (2014) apparent gaze shifts (inaccuracies) due to the PSA can be as large as 4$^\circ$. We will use the apparent gaze shift as function of pupil diameter, if present, to correct refixation positions for the refixation task. If the dispersion for the corrected refixation positions is substantially lower than for the uncorrected refixation positions, the PSA is a problem for the accuracy of pupil-CR eye trackers in general.

### Methods

#### Procedure, participants, stimulus, and task

We engaged three experienced participants (all authors on the current article; at least 18 years of eye-tracking experience) to take part in the experiment. The session started with a standard nine-point calibration and validation of the EyeLink 1000 Plus^1^.

After calibration, the refixation condition started. The visual stimulus in this experiment was a carefully chosen fixation marker (ABC in Figure 1 of Thaler et al., 2013). It was presented on a grey background and could appear on one of the nine positions of a virtual 3 x 3 grid (size 3$^\circ$ by 3$^\circ$). The outer diameter of the fixation marker was 0.7$^\circ$, and the diameter of the inner dot was 0.1$^\circ$. Participants were instructed to carefully fixate on the center (the inner dot) of the marker presented. The marker appeared at a semi-randomly chosen position from one of the eight peripheral positions of the virtual grid and remained visible for 1 s. Subsequently, the marker was displayed at the center position of the grid for 2 s. This sequence was repeated 80 times. We analyzed the fixation position on the center target.

Then, the participant was instructed to fixate on a centrally presented fixation marker on a circular grey background with a diameter of 3$^\circ$ and refrain from blinking. The rest of the screen was white for 2 s and then black for 8 s. This white-black sequence was repeated ten times to elicit pupil-size changes (total duration 100 s). We utilized the resulting pupil-size changes in conjunction with the presumed apparent shift in the gaze signal to estimate the pupil-size artefact for each participant (Wyatt, 2010). We refer to this as the PSA-estimation condition.

#### The eye-tracking setup

Gaze of the left eye was recorded by the SR Research EyeLink 1000 Plus (host software v. 5.12) at 1000 Hz (centroid pupil detection model; heuristic filters turned off; default monocular nine-point EyeLink calibration with nine-point validation). The right eye was patched. To minimize head movements, we used the standard EyeLink 1000 Plus chin and forehead rest.

#### The stimulus presentation system

The visual stimulus was presented on a 24-inch ASUS VG248QE (53.0 x 30.0 cm; 1920 pixels x 1080 pixels; 16:9 ratio; refresh rate: 60 Hz) placed at a distance of 80 cm from the eye. Stimulus presentation was done with PsychoPy version 2023.2.3 (Peirce, 2007, 2008). The light in the experimental room was turned off, however it was not completely dark because we did not intentionally shut down the 17" EyeLink control monitor.

#### Eye-tracking data processing

To detect fixations we used the Python implementation of the I2MC (v. 2.2.3) algorithm (Hessels et al., 2016). From the detected fixations, we selected the fixation with the longest duration that started at least 100 ms after onset of the central fixation target. To correct for the PSA we used a second-order polynomial fitted to the *apparent gaze shift–pupil size* relation (see Fig. 2).

### Results

#### The pupil-size artefact

To investigate how pupil size may affect accuracy, we plotted the apparent gaze shift versus pupil size. Figure 2 shows the pupil size artefact (PSA) for the horizontal (blue) and vertical (orange) component of gaze for three participants. In the case of participants P1 and P3, the pupil size range extends from approximately 3.5 mm to 6.5 mm. However, for P2, the range is smaller, from 2.5 mm to 4.5 mm. As pupil size and pupil size range decrease with age (Birren et al., 1950) and P2 is older than P1 and P3, this is to be expected. The largest apparent gaze shifts are observed in the vertical component for P1 and P3 (2.5$^\circ$ to 3$^\circ$) and in the horizontal component for P2 (1.5$^\circ$). We will later utilize this relation to correct the refixation positions for the PSA. The correction based on the PSA has been previously addressed (Drewes et al., 2014; Choe et al., 2016).

**Fig. 3:**
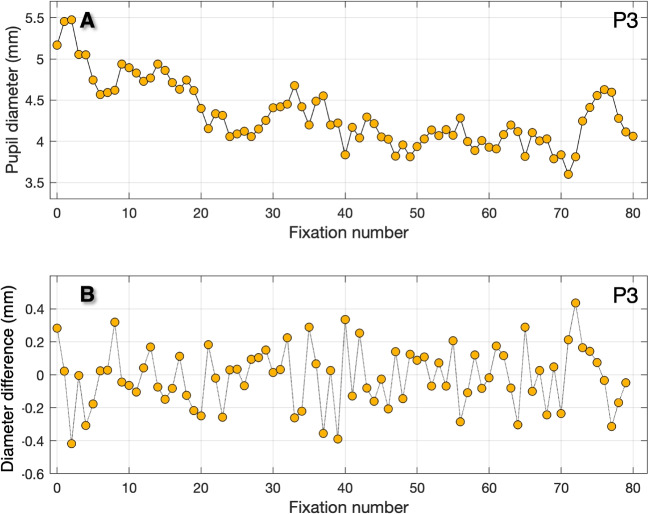
**Pupil diameter versus fixation rank number**. **A** Pupil diameter versus fixation number for P3. The pupil diameter slowly decreases from approximately 5.5 to 3.5 mm without changing the light level. **B** Pupil size change between subsequent refixations of the center target. Between subsequent refixations of the center target (3 s apart), pupil-size changes up to 0.4 mm occur

#### The pupil size as function of time

A preliminary indication that PSA may negatively impact the quality of calibration and validation is the observation that, during the refixation condition, pupil size changes significantly within the timescale of successive refixations. Figure 3 depicts the pupil diameter for all 80 refixations of the central target for P3. The pupil diameter ranges from 3.6 mm to 5.5 mm (panel A). Note, this is a range as large as evoked by a black and a white screen during the PSA-estimation condition of experiment 1 (Fig. 2C). The pupil size differences between consecutive refixations range from -0.42 mm to +0.44 mm (Fig. 3B). If we take into account the relation between pupil size and apparent gaze shift for P3 (Fig. 2C) we expect the PSA to affect inaccuracy. A rough estimate (by the slope) is that for P3 the vertical PSA is close to 1.0$^\circ$ mm$^{-1}$ (1.0$^\circ$ added inaccuracy per mm pupil-diameter change).

**Fig. 4:**
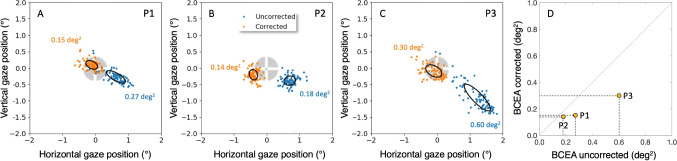
**Experiment 1. EyeLink: Refixation dispersion**. Panels **A**, **B**, and **C** show refixation positions for the uncorrected EyeLink eye-tracking data (blue) and for the PSA-corrected eye-tracking data (*orange*). The *black ellipses* on top of the refixation positions are bivariate contour ellipse areas (BCEA, Crossland and Rubin, 2002). The BCEA ellipse contains 68% of the refixations. The uncorrected data (*blue*) shows most dispersion for P1 (0.27 deg$^2$) and P3 (0.60 deg$^2$) and less for P2 (0.18 deg$^2$). For all participants, the dispersion is less for the corrected eye tracking data (*orange*): P1(0.15 deg$^2$), P3 (0.30 deg$^2$), P2 (0.14 deg$^2$). Panel **D** shows the BCEA for the corrected versus the uncorrected eye tracking data. The BCEA is, respectively, 44% (P1), 22% (P2) and 50% (P3) smaller for the corrected eye-tracking data. Note: In this experiment, we are not concerned with absolute accuracy (the distance from the center of mass of the refixations to the fixation target). The fact that the corrected data are closer to the center of the refixation target is due to our fitting procedure

#### Refixation dispersion

How well can humans refixate a visual target? In this experiment, we are not concerned with absolute accuracy (the distance from the center of mass of the refixations to the fixation target); rather, we seek to understand how closely spaced a participant’s fixations are when asked to refixate the same target repeatedly. Figure 4 shows the fixation positions (blue, labeled uncorrected) and the same refixation positions corrected for the PSA (orange). We hypothesized that the PSA would have a negative impact on fixation dispersion because nearly every fixation involves a pupil-size-related inaccuracy due to pupil size changes on a short time scale (Fig. 3). It is evident that the dispersion for all three participants is reduced after correction for PSA (Fig. 4D). We calculated the Bivariate Contour Ellipse Area (BCEA, Crossland and Rubin, 2002) for the corrected and uncorrected refixation positions. Here the BCEA is the elliptical region containing 68% of the refixations. For P1, P2 and P3, the BCEAs for the uncorrected refixation positions are 0.27 deg$^2$, 0.18 deg$^2$ and 0.60 deg$^2$ (see Table 1). For the corrected refixation positions BCEAs are 0.15 deg$^2$, 0.14 deg$^2$ and 0.30 deg$^2$. The BCEA is respectively 44% (P1), 22% (P2) and 50% (P3) smaller for the for PSA corrected fixation positions. For P2, the decrease due to correction is smaller, but this participant also had a much smaller PSA (see Fig. 2B), hence there was less room for improvement.

### Discussion Experiment 1

Based on Experiment 1, we can conclude that on the short time scale of calibration and validation, the PSA has a negative effect on the refixation dispersion (it is larger). We found that with a simple correction of the fixation positions based on the relation between apparent fixation position and pupil diameter, the dispersion decreased by tens of percent. We expect that if pupil size is incorporated as a parameter in the calibration, the accuracy of pupil-CR eye trackers can be improved significantly. The limit on pupil-CR eye tracker accuracy should not be 0.5$^\circ$. Drewes et al. (2014) have already suggested a compensation method for PSA and we will come back to this in the discussion. The conclusion of Experiment 1 is that PSA limits the accuracy of current pupil-CR eye trackers because substantial apparent gaze shifts occur on a short timescale. Whether a part of the dispersion that we obtain in the corrected refixations is due to inconsistent refixation, we do not know. To investigate this, an eye tracker is required that does not make use of the pupil to estimate gaze and preferably is more accurate and precise than an EyeLink 1000 Plus.

## Experiment 2: Fixation dispersion and retinal eye tracking

This experiment will deal with the question how well people can refixate a target. For this experiment, we chose an eye tracker, the FreezeEye Tracker, that does not use the inner iris border (the pupil) for gaze estimation, but films the retina instead.

### Methods

#### Procedure, participants, stimulus, and task

The same experienced participants from the first experiment took part in the second experiment. The session started with carefully adjusting and positioning the FreezeEye retinal eye tracker. Hereafter, the refixation condition (similar to Experiment 1) started. The only differences were the color of the fixation marker and the color of the background. The fixation marker was red on a very dark green background. We changed the target color to red to produce the sharpest image possible with this eye-tracking setup. The very dark green appeared almost black.

#### The setup

Eye tracking data was obtained utilizing the FreezeEye Tracker (FET), as outlined in Bartuzel et al. (2020). Employing the confocal scanning laser ophthalmoscope principle, this device captures small rectangular frames of the retina measuring (5.34$^\circ$ ± 0.04$^\circ$) x (2.75$^\circ$ ± 0.02$^\circ$) at a rate of 610 Hz. The trajectory of gaze is determined through pairwise alignment of these captured frames. The FET has very good precision, RMS sample-to-sample deviation measures $0.07^{\prime }$. The FET is calibration-free. We do not know the absolute accuracy of the FET. In our experiment, the zero is a choice (e.g., the first sample of the recording when the participant fixated the central fixation point). We are not interested in absolute accuracy. The aim of Experiment 2 is to determine the dispersion of the refixations of the central fixation target. Stimuli were presented by an LCD screen (16$^\circ$ x 16$^\circ$) at a rate of 90 Hz. To minimize head movements, we used a chin and forehead rest.

#### Eye tracking data processing

The fixation detection and selection method was identical to Experiment 1.

**Fig. 5:**
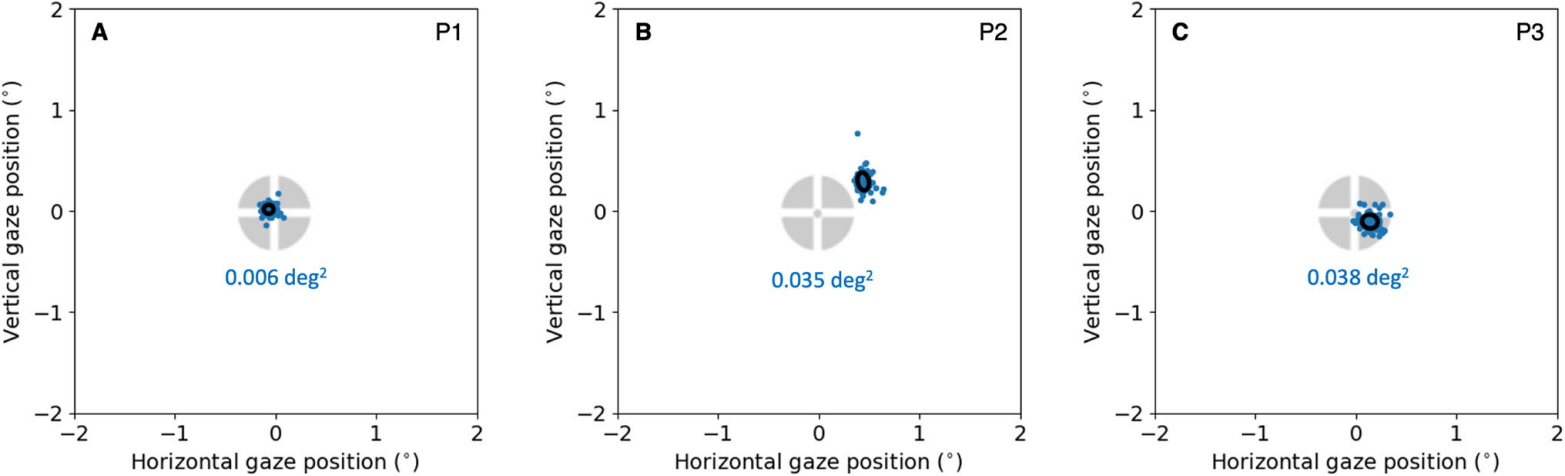
**Experiment 2. FreezeEye tracker: Refixation dispersion**. Panels **A**, **B**, and **C** show refixation positions for P1, P2 and P3. Note that the center of the collection of refixations does not necessarily coincide with the center of the fixation marker. As for many eye trackers, the zero is a choice. Here, the first sample of the recording was set to (0,0). We do this because we are only interested in the dispersion. On top of the refixation positions we plotted the BCEA ellipse (so small that it is difficult to see). The BCEA measures 0.006 deg$^2$ (P1), 0.035 deg$^2$ (P2) and 0.038 deg$^2$ (P3). For all participants, the refixation dispersion is much smaller than for the corrected fixation positions of the EyeLink 1000 plus. The BCEA is respectively 25 (P1), 4 (P2) and 8 (P3) times smaller

**Table 1:** BCEA and standard deviation of the refixations for three participants for Experiment 1 (uncorrected and corrected for PSA) and Experiment 2

	Exp 1: EyeLink uncorrected			Exp 1: EyeLink corrected			Exp 2: FreezeEye Tracker		
	BCEA (deg$^2$)	$s_{max} (^{\prime })$	$s_{min} (^{\prime })$	BCEA (deg$^2$)	$s_{max} (^{\prime })$	$s_{min} (^{\prime })$	BCEA (deg$^2$)	$s_{max} (^{\prime })$	$s_{min} (^{\prime })$
P1	0.27	19.62	6.9	0.15	11.46	6.72	0.006	2.88	2.70
P2	0.18	10.8	8.34	0.14	9.84	7.38	0.035	5.40	3.30
P3	0.60	33.06	9.24	0.30	16.02	9.54	0.038	4.74	4.08

BCEA denotes the Bivariate Contour Ellipse Area. The ellipse contains 68% of the refixations. $s_{max}$ denotes the standard deviation along the major axis and $s_{min}$ denotes the standard deviation along the minor axis of the ellipse

### Results

Figure 5 shows the refixation dispersion for three participants. Obtained BCEAs measured 0.006 deg$^2$, (P1), 0.035 deg$^2$ (P2) and 0.038 deg$^2$ (P3) (see Table 1). These BCEAs are a magnitude or more (P1) smaller than those obtained with a pupil-CR eye tracker, even after correction for PSA (see Table 1). Humans are capable of precisely refixating a visual target. Based on these results, we refute the *Inconsistent fixation* hypothesis.

## Discussion

### Summary of Results

In this study, we investigated two possible reasons behind the generally modest accuracy of 0.5$^\circ$ of most pupil-CR eye trackers. We suggested that significant variations in pupil size on a short timescale could lead to inaccuracies during both calibration and validation. Experiment 1 demonstrated substantial differences in pupil size between consecutive refixations, indicating that the PSA introduces inaccuracies up to 0.5$^\circ$ (Fig. 3) during calibration and validation. To estimate the magnitude of the negative effect of the PSA, we corrected the refixation positions for the PSA and examined by how much refixation dispersion decreased. For the two participants with a lot of variation in pupil size, the refixation dispersion was reduced by about 50% by correcting for the PSA. For the participant with less variation in pupil size (P2), the refixation dispersion was reduced by approximately 20%. We conclude that the pupil plays a big role in the inaccuracy of pupil-CR eye trackers.

Additionally, we suggested that individuals might not adequately fixate on presented visual targets during calibration. By the use of a sophisticated retinal eye tracker, Experiment 2 showed that individuals can indeed precisely refixate the center of a visual target. We found very small BCEAs (8 to 25 times smaller than the BCEAs of the PSA-corrected EyeLink fixations, see Table 1). This means that humans can precisely refixate the same target.

Together, these two experiments highlight the challenges associated with accurately determining gaze using an eye tracker based on pupil center estimates. Crane and Steele (1985) stated a long time ago that the pupil is not a stable reference, but did not explain why. We now understand the PSA occurring on a short timescale sets a limit on the accuracy of a pupil-CR eye tracker. At least experienced participants can fixate targets so well that proper refixation is not a bottleneck for achieving a high accuracy of pupil-CR eye trackers.

### How is the PSA detrimental for accuracy?

If we assume an EyeLink-like calibration–validation procedure for convenience, how does the PSA affect the accuracy during the validation process? For each validation target, the fixation offset from the target can easily be up to 0.5$^\circ$ in any direction. Accuracy is calculated as the average of the distances between the fixation points and the validation targets. In this way, an accuracy of about 0.5$^\circ$ is not unexpected.

How does the PSA affect the accuracy of the calibration? This is more indirect than during validation. The targets for calibration are typically positioned at a large distance from each other (for example, more than 10$^\circ$). An apparent gaze shift caused by a larger or smaller pupil (up to 0.5$^\circ$) will have an effect on the fit parameters of the polynomial or the eye model. When the calibration targets are far apart, we do not expect a very large effect from the PSA, because the apparent gaze shift is small compared to the distance between the calibration targets. We expect the PSA to have a larger effect on the accuracy of the parameter fit if the calibration grid is small (e.g., 5$^\circ$ x 5$^\circ$).

Some researchers repeat a calibration-validation cycle until the reported mean accuracy falls below a certain threshold. We assume they want to achieve the highest accuracy possible. Two examples are Foulsham et al. (2008) and Tatler (2007). With the knowledge from the current study, we understand that the accuracy value for participants with a significant apparent gaze shift could appear worse, and the accuracy value may have little relevance to the “true" accuracy. Repeating a calibration until the accuracy falls under 0.5$^\circ$ will not make the eye tracking data quality better. In our opinion, any accuracy value of the order to 0.5$^\circ$ is good enough. If accuracy turns out to be poor (e.g., if the participant did not look at the correct point(s)), recalibration is always warranted. One message of this article is that accuracy is dynamic and depends on the idiosyncratic PSA (Drewes et al., 2014; Hooge et al., 2021) and the fluctuation in the size of the pupil (as in Fig. 3B). The elderly usually have smaller pupils (Birren et al., 1950) and we expect that for them PSA is less a problem than for the accuracy of the eye tracking data of younger individuals because expected pupil size changes are smaller. Elderly individuals typically have smaller pupils and a smaller range of pupil size compared to younger individuals (Birren et al., 1950). When the range of pupil size changes is smaller, the apparent gaze shifts due to the PSA also becomes smaller. Therefore, PSA poses less of a problem for the accuracy of eye-tracking data in older individuals compared to younger ones.

### Correcting for the PSA

In their innovative article, Drewes et al. (2014) suggested three methods to compensate for the PSA in pupil-CR eye trackers. In the first method (two-point) they proposed to perform two separate calibrations, one with a dark background and one with a light background. This way, they calibrated with an assumed large pupil (dark background) and with an assumed small pupil (light background), allowing, for instance, correction for the PSA with an interpolation method. The second method (three-point) is similar to the first method, the difference is that a third intermediate background is added, allowing for a quadratic interpolation method. However, our Fig. 3A shows that on the timescale of a calibration (e.g., 9 fixations), significant variations in pupil size may occur between different fixations even without changes in background brightness. The two- and three-point methods of Drewes et al. (2014) do not account for light-level independent pupil-size variability during a nine-point calibration. Their third method, to which they refer as “Look Up Table" (LUT), is comparable to our method in the sense that it concerns “mapping pupil size to drift magnitude" (p. 4). What they refer to as *drift* we call *apparent gaze shift*. Drewes et al. (2014) also for warn for hysteresis. Based on our Experiment 1, we would advise using the third method. Here we want to propose a new calibration method.

We propose a *pupil size - apparent shift* characteristic generated at each calibration location (e.g., 9 as in the standard EyeLink method) as shown in Fig. 2. This method is insensitive to additional changes in pupil size due to causes other than changes in light intensity. However, the proposed method has a significant drawback–it is very time-consuming. When repeating a calibration with nine points, each with ten cycles of dark-bright variations that take 10 s, a calibration takes in total 9 x 10 x 10 min = 900 s (15 min). Which experiments warrant such an investment? Perhaps experiments where high accuracy is crucial (e.g., reading small letters or studies with small AOIs) and the study of vergence (Hooge et al., 2019).

### Implications for research with pupil-CR eye trackers

We do not want to appear alarmist in this article. An accuracy of 0.5$^\circ$ may pose no hindrance to conducting a successful eye-tracking study. This is particularly applicable to studies involving AOIs of sufficient size (e.g., Holmberg et al., 2014; van der Laan, 2015; Vehlen et al., 2022). See Holmqvist et al. (2012) for an explanation of the relation between accuracy and AOI size.

### How well can humans refixate a target?

How good are the dispersion values of Experiment 2? We could compare them with the values from Poletti and Rucci (2016). In their Figure 3, they present dispersion values for fixations on calibration targets at various locations on the screen measured with a Dual Purkinje Image (DPI) tracker. This is not precisely the same as what we did in the refixation condition, but it is quite similar. Poletti and Rucci (2016) distinguished between standard calibration (participant presses a button when the target is fixated) and manual calibration, which includes a gaze-contingent manual offset correction. The standard calibration method delivered a horizontal standard deviation of ${3.8}^{\prime }$ and a vertical standard deviation of ${6.3}^{\prime }$. The gaze-contingent manual offset correction method yields the best (smallest) values for dispersion, namely a horizontal standard deviation of ${1.9}^{\prime }$ and a vertical standard deviation of ${2.4}^{\prime }$. In our study, we determined the BCEA (the elliptical area yielding 68% of the refixations) and the standard deviation along the major ($s_{max}$) and minor ($s_{min}$) axis of the ellipse. $s_{max}$ and $s_{min}$ range from ${2.7}^{\prime }$ to ${5.4}^{\prime }$ (see Table 1). The values for *s* reported from Experiment 2 fall in the range reported by Poletti and Rucci (2016).

## Conclusion

Pupil size changes in combination with the PSA are an important factor in the modest accuracy of 0.5$^\circ$ of pupil-CR eye trackers. Humans are excellent at refixating visual targets, so this ability does not limit the accuracy of pupil-CR eye trackers.

## Data Availability

Due to data protection rules, the data collected in Experiments 1 and 2 cannot be made available.

## Footnotes

1 We only make one calibration despite assuming that refixation error and PSA may be present in the calibration phase as well. This is because we do not care about minimizing the calibration error (absolute error or accuracy) but only want to quantify the dispersion (relative error) between refixations.

## Code availability

Documented code for the analyses in this article is available at https://osf.io/pvhdg and is illustrated with a separate recording made for illustrative purposes.

## Declarations

### Open Practices Statement

There are no data associated with the present article.
